# Lower‐Cost Nonsurgical Primary Prevention Strategies for Women at High Risk of Breast Cancer: A Scoping Review

**DOI:** 10.1155/tbj/1523594

**Published:** 2026-10-01

**Authors:** Menghua Wen, Youfu Zhao, Chunying He

**Affiliations:** ^1^ Yan’an Medical College Yan’an University Yan’an Shaanxi China yau.edu.cn; ^2^ Medical Oncology Affiliated Hospital of Yan’an University Yan’an Shaanxi China yau.edu.cn

**Keywords:** breast cancer, cost, nonsurgical treatment, primary prevention, scoping review

## Abstract

**Background:**

A systematic mapping of nonsurgical, lower‐cost primary prevention strategies for women at high risk of breast cancer remains limited.

**Objective:**

To identify and summarize lower‐cost (i.e., lower economic cost relative to surgical and pharmaceutical alternatives; not dependent on expensive equipment, patented drugs, or complex medical procedures; implementable through community, family, or self‐management models), nonsurgical primary prevention strategies for women at high risk of breast cancer, providing empirical evidence to inform clinical practice, health education, and public health policy.

**Methods:**

Following the Joanna Briggs Institute (JBI) methodology for scoping reviews and the Participants, Concept, and Context (PCC) framework, we systematically searched 9 Chinese and English databases. Search terms covered “breast cancer,” “high‐risk women,” “primary prevention,” and various nonsurgical strategies. Two independent reviewers performed literature screening and data extraction.

**Results:**

Seventeen studies were included, and six prevention strategy categories were identified: nutritional dietary interventions, physical activity interventions, comprehensive lifestyle interventions, chemoprevention, digital health interventions, and health education with self‐management. Comprehensive lifestyle intervention emerged as the core strategy with robust evidence and manageable costs. Nutritional interventions have shifted from single nutrients to overall dietary patterns. Chemoprevention, limited by cost and side effects, is more suitable for very high‐risk women. Digital health interventions and health education offer advantages in medically underserved areas.

**Conclusion:**

This scoping review systematically maps lower‐cost, nonsurgical primary prevention strategies for women at high risk of breast cancer, identifying six categories of promotable interventions. Comprehensive lifestyle intervention represents the most evidence‐supported and economically manageable core strategy. For the first time from the dual perspectives of “nonsurgical” and “lower cost,” this review provides practical references for primary healthcare systems, clinical education, and public health policy, with particular relevance for resource‐limited settings.

## 1. Introduction

Breast cancer is among the most frequently diagnosed malignancies in women globally, with its incidence surpassing that of lung cancer to rank first worldwide [1]. According to global statistics on new cancer cases, approximately 2.3 million women were diagnosed with breast cancer in 2020, representing a major threat to women’s health and a persistent, serious challenge to public health systems [1]. Although the exact etiology of breast cancer remains incompletely understood, a broad consensus has been reached regarding several risk factors [2], including family history, genetic mutations, benign breast disease, lifestyle factors, hormone replacement therapy, age, reproductive history, and diabetes, all of which have been closely linked to breast cancer development [3, 4]. In this context, prevention strategies must shift from postdiagnosis treatment to prediagnosis primary prevention, an inevitable step in addressing the global burden of breast cancer. Although therapeutic advances have improved outcomes for breast cancer patients [5], primary prevention for high‐risk women remains an indispensable component of disease control strategies and should be advanced in parallel with treatment to reduce the incidence of breast cancer.

For women carrying risk factors, prophylactic mastectomy represents the most effective option for risk reduction [6]; however, its invasiveness, potential negative impact on quality of life, and high costs preclude its use as a universal public health strategy [7, 8]. Current research has largely focused on the financial toxicity associated with breast cancer, including treatment costs, psychological burden, out‐of‐pocket expenses, transportation, and accommodation with few studies addressing primary prevention strategies prior to disease onset [9, 10]. In contrast, studies specifically addressing primary prevention strategies for high‐risk women prior to breast cancer onset remain relatively limited [11]. Existing reviews have largely focused on single risk factors or interventions, lacking systematic integration across multiple strategies [3]. This gap leaves public health policy without economically cost‐effective, universally applicable preventive options beyond surgery for large populations of high‐risk women.

Existing primary prevention strategies for women at high risk of breast cancer primarily include lifestyle interventions (e.g., dietary modification, physical activity, weight management), chemoprevention (e.g., selective estrogen receptor modulators and aromatase inhibitors), and prophylactic surgery [3]. While highly effective, prophylactic surgery is limited by its invasiveness and cost [8]. Chemoprevention, despite its efficacy, is constrained by side effects and long‐term adherence issues [12]. Lifestyle interventions are generally safe and have potential cost‐effectiveness [13], but existing reviews predominantly focus on single behavioral changes (e.g., diet alone or exercise alone) [14, 15], and few provide systematic integration across multiple strategy categories (e.g., dietary, physical activity, chemoprevention, digital health) within a single synthesis [16]. Moreover, current reviews tend to concentrate on the effects of single interventions or on secondary prevention [11]; a systematic synthesis of breast cancer primary prevention strategies from the dual perspectives of “nonsurgical” and “lower‐cost” remains absent.

Primary prevention refers to proactive measures targeting the causes or risk factors of a disease before its onset [3, 4]. The distinctive feature of this study is its focus on nonsurgical prevention strategies for women at high risk of breast cancer, aimed at modifiable risk factors, lifestyle habits, and enhancing individual resilience, thereby reducing breast cancer risk and improving quality of life.

Accordingly, this scoping review aims to comprehensively search and map existing evidence to distribution and characteristics of lower‐cost (i.e., lower economic cost relative to surgical and pharmaceutical alternatives; not dependent on expensive equipment, patented drugs, or complex medical procedures; implementable through community, family, or self‐management models), nonsurgical prevention strategies for high‐risk women, from the perspectives of public health and health equity [17, 18]. This framework is intended to provide directly actionable scientific guidance for health education promotion in community and primary healthcare settings, as well as a reference for clinical practitioners conducting patient health education.

## 2. Materials and Methods

### 2.1. Defining the Research Question

Through a systematic synthesis of the available literature, this study formulated the following research question: What lower‐cost, nonsurgical primary prevention strategies are available for women at high risk of breast cancer?

### 2.2. Literature Search Strategy

English‐language publications were searched in PubMed, Web of Science, EMBASE, Cochrane Library, and CINAHL. Chinese‐language publications were searched in the China National Knowledge Infrastructure (CNKI), Wanfang Database, VIP Database, and China Biomedical Literature Database (CBM). A combination of controlled vocabulary (subject headings) and free‐text terms was employed. The search period covered from database inception to November 30, 2025. Reference lists of included studies were also manually traced for additional relevant publications. The search strategy was developed by the first author under the guidance of a researcher with methodological expertise in systematic reviews, and refined through iterative pilot searches and team discussions. The search formulas are presented in Supporting Information 1.

### 2.3. Inclusion and Exclusion Criteria

Inclusion criteria were defined following the Joanna Briggs Institute (JBI) Participants, Concept, and Context (PCC) framework outlined in the methodological guidance for scoping reviews. (1) Participants (P): women explicitly identified or described as being “at high risk for breast cancer” in the original studies, aged 18 years or older. High‐risk status was determined based on the original descriptions, including but not limited to: family history of breast cancer; carriage of known breast cancer susceptibility gene mutations (e.g., BRCA1/2); history of benign breast disease (e.g., atypical hyperplasia); long‐term hormone replacement therapy; high breast density; and health conditions or lifestyle factors associated with breast cancer risk, such as diabetes and obesity. (2) Concept (C): nonsurgical primary prevention strategies adopted by high‐risk women prior to breast cancer onset. (3) Context (C): primary prevention of breast cancer.

Exclusion criteria were as follows: (1) studies involving patients already diagnosed with breast cancer or any other malignancy; (2) studies investigating breast cancer risk factors, pathogenesis, or risk prediction, or those focusing on breast cancer treatment, rehabilitation (including reconstructive surgery), or prognosis (e.g., infections, complications); (3) studies focusing on secondary prevention (e.g., screening) or tertiary prevention (e.g., recurrence prevention); (4) basic or translational research focused on disease biology, diagnostic techniques, or therapeutic targets (e.g., frozen section analysis, cell experiments, pathological characterization); (5) intervention studies relying on advanced medical equipment, complex in‐hospital procedures, or highly expensive patented drugs (e.g., protocols using advanced imaging); (6) duplicate publications; (7) studies with unavailable full text; (8) nonoriginal research (e.g., conference abstracts, case reports, case series, comments, expert opinions); and (9) studies not published in Chinese or English.

### 2.4. Literature Screening and Data Extraction

All retrieved records were imported into EndNote 2025 for duplicate removal. Two trained graduate students then independently screened the titles and abstracts against the inclusion and exclusion criteria to exclude clearly ineligible studies. Full texts of potentially eligible studies were subsequently retrieved and independently assessed in a second round of screening. Disagreements during the screening process were resolved by inviting a third researcher to participate in discussion until consensus was reached. The following data were extracted from each included study: first author, country, study type, publication year, sample size, risk factors as explicitly defined in the original study, preventive measures, key findings/conclusions, and outcome measures. This study has been registered on the Open Science Framework (OSF; registration number: https://doi.org/10.17605/OSF.IO/TS5Q6) and reported in accordance with the Preferred Reporting Items for Systematic Reviews and Meta‐Analyses extension for Scoping Reviews (PRISMA‐ScR) checklist. The checklist is provided in Supporting Information 2.

### 2.5. Quality Assessment

All included studies underwent quality assessment to provide a descriptive overview of the methodological quality of the included evidence. Randomized controlled trials (RCTs) [19–22] were evaluated using the Cochrane Risk of Bias 2.0 (RoB 2.0) tool. Cohort and case–control studies [17, 18, 22–29] were assessed using the Newcastle–Ottawa Scale (NOS). The quasi‐experimental and single‐arm studies [30, 31] were evaluated using the methodological index for nonrandomized studies (MINORS) checklist. The qualitative interview study [32] was assessed using the Joanna Briggs Institute Qualitative Assessment and Review Instrument (JBI‐QARI) checklist, and the cross‐sectional study [33] using the JBI standard checklist.

## 3. Results

### 3.1. Literature Screening Results

The systematic search yielded 6372 records. After duplicate removal using EndNote 2025, 5670 records remained. Two independent reviewers screened the literature against the inclusion and exclusion criteria. Title and abstract screening excluded 5079 irrelevant records, leaving 591 records for full‐text assessment. Full‐text reading led to the exclusion of an additional 575 records, leaving 16 records. One further record was identified through reference tracing, bringing the final number of included studies to 17. The literature screening process is presented in Figure 1.

**FIGURE 1:**
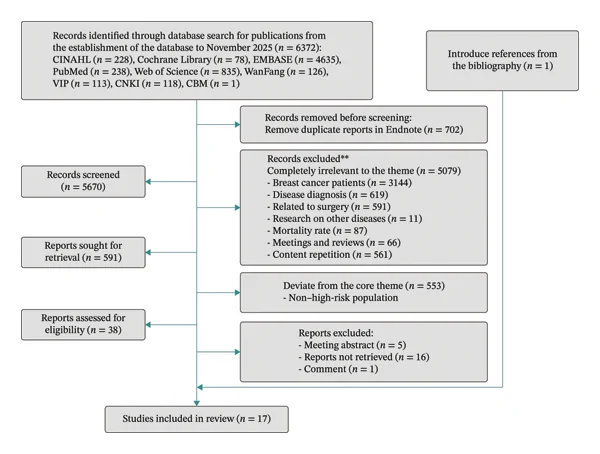
The flowchart for literature screening.

### 3.2. Basic Characteristics of Included Studies

Of the 17 included studies, the countries of origin were the United States (*n* = 8) [17–22, 24, 31], Korea (*n* = 2) [28, 29], the United Kingdom (*n* = 2) [26, 32], Spain (*n* = 1) [23], China (*n* = 1) [30], France (*n* = 1) [25], Lebanon (*n* = 1) [33], and a multicenter European study (*n* = 1) [27]. All included studies were published between 2014 and 2025. Sample sizes ranged from 13 to 4,841,083, with a median of 3614. The majority of studies employed a cohort design (*n* = 8) [18, 23–29], followed by RCTs (*n* = 3) [19–21], a quasi‐experimental study (*n* = 1) [30], a casecontrol study (*n* = 1) [17], a study incorporating both cohort and RCT designs (*n* = 1) [22], a cross‐sectional survey (*n* = 1) [33], a qualitative interview study (*n* = 1) [32], and a single‐arm study (*n* = 1) [31]. Regarding preventive focus, seven studies addressed dietary prevention [17, 19, 23–25, 28, 29], five focused on comprehensive lifestyle prevention (e.g., healthy diet, physical activity, positive lifestyle habits and mindset) [20, 22, 27, 30, 33], two on digital behavioral interventions [21, 32], two on chemoprevention [26, 31], and one on physical activity alone [18]. The basic characteristics of the included studies are summarized in Table 1.

**TABLE 1:** Basic characteristics of included studies.

First author/year	Country	Design	Sample size/cases	Risk factors	Preventive measures	Breast cancer–related outcomes	Key findings
Peng et al. [17], 2021	USA	Case–control	3614/1919	Genetic susceptibility, high mammographic density, early menarche, nulliparity, late first childbirth, family history, benign breast disease, alcohol, physical inactivity, poor diet, smoking, postmenopausal status	Increase circulating carotenoids; consume carotenoid‐rich foods (carrots, citrus, tomatoes, leafy greens)	Breast cancer risk	Higher circulating carotenoids significantly reduce risk in genetically susceptible or high‐density women. Diet alone showed no clear association, possibly due to absorption or measurement issues.
Egnell et al. [25], 2017	France	Prospective cohort	27,853	Higher alcohol intake (≥ 3.6 g/day), family cancer history, taller height, secondary education	B vitamin supplements (pyridoxine, thiamine)	Breast cancer risk	B vitamins may protect against breast cancer in middle‐aged women, but only in low/nondrinkers; alcohol may interfere with absorption/metabolism.
Katsaroli et al. [18], 2024	USA	Retrospective cohort	44,463/994	Age, genetics, weight gain, sedentary behavior, poor diet (red meat, sugar, processed foods), alcohol, low CRF	(1) ≥ 150 min/week moderate aerobic exercise to improve CRF; (2) Comprehensive lifestyle intervention (weight control, balanced diet, smoking/alcohol cessation)	Breast cancer incidence	CRF is inversely associated with breast cancer risk (dose–response). Each 1‐MET increase reduces risk by 7%. Improving CRF lowers risk.
Llanos et al. [19], 2014	USA	RCT	70	Postmenopausal, BMI 25.0–42.0, first‐degree family history	(1) Tomato‐based diet; (2) Soy protein products	Serum adipokines (adiponectin, leptin)	Tomato intake may raise adiponectin (potentially protective); soy may lower leptin (potentially protective). Long‐term effects require further study.
Hawkes et al. [32], 2024	UK	Qualitative interview	20	Family history, overweight/obesity, alcohol, smoking, physical inactivity	2‐month digital intervention: video session, health education micro‐website, private Facebook group	Qualitative data on acceptability, behavior change, app feedback, peer support needs	Digital interventions combining education, tracking, and peer support are acceptable for managing health behaviors and preventing weight gain. Personalization by age (18–35 years) is needed.
Romanos‐Nanclares et al. [23], 2021	Spain	Prospective cohort	4,841,083	High intake of unhealthy plant‐based foods (juices, sugary drinks, refined grains, potatoes, sweets)	Increase healthy plant‐based foods (whole grains, fruits, vegetables, nuts, legumes, vegetable oils, tea/coffee); reduce unhealthy plant‐based and animal‐based foods	Breast cancer risk	A healthy plant‐based diet significantly reduces breast cancer risk.
Puri et al. [31], 2025	USA	Single‐arm study	51	5‐year Gail risk ≥ 1.7%, LCIS/resected DCIS, ADH, ALH, BRCA1/2/PTEN/TP53 mutations, high BRCA probability, prior breast cancer (remission ≥ 5 years) with unirradiated breast	Oral hydroxytyrosol (HT) 25 mg/day for 1 year (no standard anti‐estrogens)	Change in breast density	HT did not significantly reduce breast density overall, but significant reduction was observed in women aged ≥ 60 years with high baseline density.
Romanos‐Nanclares et al. [24], 2022	USA	Prospective cohort	169,973	High‐insulin diet (processed meat, red meat, high/low‐energy beverages), obesity, weight gain	Reduce high‐insulin foods; increase low‐insulin foods (whole fruits, leafy greens, full‐fat dairy, coffee); physical activity for weight control	Invasive breast cancer incidence	High‐insulin dietary pattern (highest EDIH quintile) is significantly positively associated with invasive breast cancer risk.
Han et al. [20], 2017	USA	RCT	13/6	Gail risk ≥ 1.7% (5y), Claus/IBIS lifetime risk > 20%, ADH/ALH/LCIS, treated DCIS, BRCA1/2 mutations, high BRCA probability, BMI 25–45 (pre) or 28–40 (post)	6‐month DEEM intervention (diet, exercise, emotion, mindfulness) based on social cognitive theory; includes psychoeducation, group sessions, motivational enhancement, CBT	Body composition, biomarkers (leptin, adiponectin, insulin, glucose, CRP, IL‐6), diet, physical activity	DEEM significantly improved weight, body fat percentage, and metabolic biomarkers (leptin, adiponectin, insulin, glucose, CRP, IL‐6) in high‐risk women.
Pegington et al. [26], 2019	UK	Prospective cohort	136	Family history, overweight/obesity, alcohol, smoking, inactivity, CVD/metabolic syndrome markers	Tamoxifen (20 mg/day) for 1 year + weight control, alcohol restriction, increased physical activity	Body composition; breast cancer/CVD/metabolic syndrome markers; lifestyle adherence	Tamoxifen plus lifestyle improvements significantly reduces breast cancer and other noncommunicable diseases in high‐risk women.
Vasson et al. [27], 2025	Multi‐center Europe (10 countries)	Prospective cohort	125,746/2175	Smoking, alcohol, increased BMI, physical inactivity	Lifestyle modification	Invasive breast cancer incidence	Moderate lifestyle changes (smoking, alcohol, activity) were not associated with lower postmenopausal risk, but BMI improvement was significantly associated with lower risk.
Marinac et al. [21], 2018	USA	RCT	55/28	Sedentary (< 60 min/week moderate activity), family history, ≤ 3 servings fruits/vegetables daily	Internet‐based physical activity intervention (TTM/SCT): 45–60 min/week moderate‐vigorous activity, self‐monitoring, progress charts, peer interaction, education, psychologist calls, 2–3 personalized motivational emails/week	Physical activity; self‐efficacy	Self‐efficacy and cognitive behavioral processes are key targets for internet‐based physical activity interventions in women with a family history of breast cancer.
Shin et al. [28], 2019	Korea	Prospective cohort	78,320/359	Age, education, age at first childbirth, parity, menarche, oral contraceptives, exercise, alcohol, smoking, marital status, menopause, family history	Increased milk intake (women < 50 years)	Breast cancer incidence	Higher milk intake reduces breast cancer risk in Korean women ≤ 50 years; yogurt/cheese show no significant association.
Shin et al. [29], 2020	Korea	Prospective cohort	78,320/359	White rice dietary pattern (high white rice/salted fish, low multigrain rice/tubers/legumes/tofu/nuts/milk), age, hormonal status	Increased multigrain rice or whole‐grain products (women < 50 years)	Breast cancer incidence	White rice pattern is positively associated with breast cancer risk. In women < 50 years, higher multigrain/whole‐grain intake reduces risk.
Deeb et al. [33], 2024	Lebanon	Cross‐sectional	25	Age > 50 years, weight gain, overweight/obesity, menopause, high processed/red meat, low fruits/vegetables/fish/dairy, family history, negative nutrition attitudes	Increase fruits/vegetables (≥ 400 g/day), fish (≥ 180 g/week), dairy (≥ 2 servings/day); reduce processed meat (< 21 g/week) and red meat (≤ 500 g/week); healthy weight; no smoking/excess alcohol; positive nutrition awareness	KAP (knowledge, attitudes, practices)	Improving KAP regarding breast cancer risk promotes healthy dietary habits and reduces risk.
Pan et al. [22], 2019	USA	Observational + RCT	Observational: 61,335; RCT: 48,835	Weight gain (≥ 5%), obesity (BMI ≥ 30 kg/m^2^)	Weight loss (≥ 5%), low‐fat diet (more fruits, vegetables, grains)	Breast cancer incidence and mortality	Weight gain (≥ 5%) increases triple‐negative breast cancer risk; obesity increases risk; weight loss (≥ 5%) reduces risk; low‐fat diet reduces postdiagnosis mortality.
Zhang [30], 2025	China	Quasi‐experimental	82/41	Genetic, physicochemical, endocrine, abnormal lifestyle, obesity, mental stress, alcohol, smoking	Primary prevention risk model; diversified health education; lifestyle guidance (sleep, emotions, diet, exercise, weight); breast self‐examination training; precancerous lesion follow‐up	Self‐care ability scores (reproductive‐age women; ESCA)	A primary prevention risk assessment model improves women’s risk awareness and self‐care ability, supports clinical primary prevention, and increases voluntary screening rates.

*Note:* Multicenter Europe (10 countries): Denmark, France, Germany, Greece, Italy, Netherlands, Norway, Spain, Sweden, and the United Kingdom. BRCA1/2, breast cancer susceptibility genes 1 and 2; CRF, cardiorespiratory fitness; CVD, cardiovascular disease; DEEM, Diet, Exercise, Emotion processing, and Mindfulness; EDIH, Empirical Dietary Inflammatory Index; IL‐6, interleukin‐6; PTEN, phosphatase and tensin homolog; TP53, tumor protein p53; TTM, transtheoretical model.

Abbreviations: ADH, atypical ductal hyperplasia; ALH, atypical lobular hyperplasia; BMI, body mass index; CBT, cognitive behavioral therapy; CRP, C‐reactive protein; DCIS, ductal carcinoma in situ; ESCA, Exercise of Self‐Care Agency; KAP, knowledge attitudes and practices; LCIS, lobular carcinoma in situ; MET, metabolic equivalent of task; RCT, randomized controlled trial; SCT, social cognitive theory; UK, the United Kingdom; USA, the United States of America.

### 3.3. Quality Assessment Results

Of all included studies, 12 were rated as high quality [17, 18, 22–29, 32, 33] and 6 as moderate quality [19–22, 30, 31]. No studies were classified as low quality. It should be noted that Pan et al. [22] encompassed two study designs (a prospective cohort and an RCT) and was therefore evaluated separately for each. The quality assessment results are presented in Table 2 and Supporting Information 3.

**TABLE 2:** Quality assessment of included studies.

First author/year	Country	Study design	Sample size	Assessment tool	Raw score	Quality rating
Peng et al. [17], 2021	USA	Case–control	3614	NOS	7	High
Egnell et al. [25], 2017	France	Prospective cohort	27,853	NOS	9	High
Katsaroli et al. [18], 2024	USA	Retrospective cohort	44,463	NOS	9	High
Llanos et al. [19], 2014	USA	RCT	70	RoB 2.0	Some concerns	Moderate
Hawkes et al. [32], 2024	UK	Qualitative interview	20	JBI‐QARI	A	High
Romanos‐Nanclares et al. [23], 2021	Spain	Prospective cohort	4,841,083	NOS	9	High
Puri et al. [31], 2025	USA	Single‐arm (no control)	51	MINORS	11	Moderate
Romanos‐Nanclares et al. [24], 2022	USA	Prospective cohort	169,973	NOS	9	High
Han et al. [20], 2017	USA	RCT	13	RoB 2.0	Some concerns	Moderate
Pegington et al. [26], 2019	UK	Prospective cohort	136	NOS	8	High
Vasson et al. [27], 2025	Multi‐center Europe^1^	Prospective cohort	125,746	NOS	9	High
Marinac et al. [21], 2018	USA	RCT	55	RoB 2.0	Some concerns	Moderate
Shin et al. [28], 2019	Korea	Prospective cohort	78,320	NOS	8	High
Shin et al. [29], 2020	Korea	Prospective cohort	78,320	NOS	8	High
Deeb et al. [33], 2024	Lebanon	Cross‐sectional survey	25	JBI standard checklist	8	High
Pan et al. [22], 2019 (OS)^2^	USA	Prospective cohort	61,335	NOS	9	High
Pan et al. [22], 2019 (DM)^2^	USA	RCT	48,835	RoB 2.0	Some concerns	Moderate
Zhang [30], 2025	China	Quasi‐experimental (with control)	82	MINORS	17	Moderate

*Note:* Quality ratings were uniformly converted to three levels (high, moderate, low) according to the following criteria. RoB 2.0 for RCTs: Low risk = high, some concerns = moderate, high risk = low. NOS for cohort/case–control studies: 7–9 = high, 5–6 = moderate, 0–4 = low. MINORS for single‐arm/quasi‐experimental studies: without control (13–16 = high, 9–12 = moderate, 0–8 = low); with control (19–24 = high, 13–18 = moderate, 0–12 = low). JBI standard checklist for cross‐sectional studies: 7–8 “yes” = high, 4–6 “yes” = moderate, 0–3 “yes” = Low. JBI‐QARI for qualitative studies: Grade A = high, Grade B = moderate, Grade C = low. DM = dietary modification trial (RCT component of the same publication).

Abbreviations: RCT, randomized controlled trial; UK, the United Kingdom; USA, the United States of America.

^1^Denmark, France, Germany, Greece, Italy, Netherlands, Norway, Spain, Sweden, and the United Kingdom.

^2^OS = observational study.

### 3.4. Synthesis and Classification of Breast Cancer Risk Factors

Risk factors reported across the 17 included studies were synthesized and classified into four categories: nonmodifiable factors, modifiable lifestyle factors, reproductive and hormonal factors, and metabolic and health status factors. (1) Nonmodifiable factors included family history of breast cancer, carriage of breast cancer susceptibility gene mutations (e.g., BRCA1/2), advanced age, high breast density, and personal history of benign breast disease (e.g., atypical hyperplasia, lobular carcinoma in situ). (2) Modifiable lifestyle factors included overweight or obesity, physical inactivity, unhealthy dietary patterns (e.g., high‐insulin diet, excessive refined grain intake), alcohol consumption, and smoking. (3) Reproductive and hormonal factors included menopausal status, early age at menarche, late age at first childbirth, and history of hormone replacement therapy. (4) Metabolic and health status factors included abnormalities in metabolic syndrome‐related indicators (e.g., hyperglycemia, dyslipidemia), cardiovascular disease risk factors, and diabetes.

Notably, considerable heterogeneity existed across studies in the criteria used to define “high risk.” Some studies employed the Gail model score (≥ 1.7%), some relied on family history or genetic mutations, some on breast pathological diagnoses (e.g., atypical hyperplasia), and others on metabolic status or lifestyle characteristics. This lack of standardized definition limits cross‐study comparability and data integration.

### 3.5. Synthesis and Summary of Lower‐Cost, Nonsurgical Primary Prevention Strategies for Breast Cancer

Based on a systematic mapping of 17 studies, nonsurgical primary prevention strategies for women at high risk of breast cancer were classified into six major categories: nutritional dietary interventions, physical activity interventions, comprehensive lifestyle interventions, chemoprevention, digital health interventions, and health education with self‐management. Each category is detailed below.

### 3.6. Nutritional Dietary Interventions

Nutritional dietary intervention was the most frequently reported prevention strategy among the included studies (*n* = 7). Available evidence can be summarized into two main directions: the establishment of overall dietary patterns and the supplementation of specific nutrients.

Regarding the establishment of overall dietary patterns, evidence from large‐scale prospective cohort studies demonstrates that a healthy plant‐based diet‐rich in whole grains, fruits, vegetables, nuts, legumes, and vegetable oils significantly reduces breast cancer risk, whereas an unhealthy plant‐based diet (e.g., nonfresh fruit juices, sugar‐sweetened beverages, refined grains) shows a positive association with risk [25]; a high‐insulin dietary pattern (e.g., processed meats, red meat, high‐energy and low‐energy beverages) is similarly associated with increased breast cancer risk [21]. With respect to dairy and grains, women under 50 years of age who increase milk intake [32] or adopt multigrain rice and whole‐grain dietary patterns may reduce breast cancer risk, while a white rice‐based dietary pattern is positively associated with risk [26].

Regarding the supplementation of specific nutrients, available evidence suggests that carotenoids [19], B vitamins [27], and tomato and soy protein [24] show varying degrees of protective effects across different populations. However, the effectiveness of these nutrient supplements is modified by individual baseline characteristics such as genetic susceptibility, mammographic density, alcohol consumption, menopausal status, and obesity. Elevated circulating carotenoid levels significantly reduce breast cancer risk among women with genetic susceptibility or high mammographic density [19]; the protective effect of B vitamins (e.g., pyridoxine and thiamine) against breast cancer risk in middle‐aged women is more pronounced among low or nondrinkers, with no association observed in heavy drinkers, possibly due to alcohol interference with B vitamin absorption and metabolism [27]; among nonobese postmenopausal women, increased tomato and soy (isoflavone) intake may raise serum adiponectin levels and reduce leptin levels, respectively, thereby exerting a potential preventive effect through adipokine modulation [24]. The above evidence indicates that the establishment of overall dietary patterns yields relatively stable protective effects across populations, whereas the effectiveness of specific nutrient supplementation is significantly influenced by individual baseline characteristics.

### 3.7. Physical Activity and Comprehensive Lifestyle Interventions

Physical activity represents a common and modifiable lifestyle factor for women at high risk of breast cancer. Katsaroli et al. [18] identified a significant negative dose–response relationship between cardiorespiratory fitness (CRF) and breast cancer risk: each 1‐metabolic equivalent increase in CRF corresponded to a 7% reduction in risk (HR = 0.93, 95% CI: 0.90–0.95). This study employed standardized exercise testing to objectively assess CRF, and the results showed that higher CRF was negatively associated with breast cancer risk.

Comprehensive lifestyle intervention is a multidimensional behavior change strategy addressing diet, physical activity, weight management, and psychological well‐being. Han et al. [20] implemented a 6‐month DEEM intervention (diet, exercise, emotion processing, and mindfulness) grounded in Bandura’s social cognitive theory (SCT). Through psychoeducation, group discussions, and motivational enhancement, the intervention significantly improved body weight, body fat percentage, and metabolic biomarkers including leptin, adiponectin, insulin, and glucose. Vasson et al. [27] found that midlife body mass index (BMI) improvement was associated with reduced breast cancer risk, but moderate lifestyle modifications (smoking, alcohol intake, physical activity) showed no significant association with postmenopausal breast cancer risk. Pan et al. [22] demonstrated that weight loss of ≥ 5% significantly reduced breast cancer risk, and a low‐fat dietary pattern significantly reduced post–breast cancer mortality. Deeb et al. [33] reported that among women aged > 50 years with weight gain or overweight/obesity, increasing intake of fruits, vegetables (≥ 400 g/day), fish (≥ 180 g/week), and dairy products (≥ 2 servings/day), reducing processed meats (< 21 g/week) and red meat (≤ 500 g/week), maintaining healthy weight, avoiding smoking and excessive alcohol use, and enhancing awareness of the nutrition–breast cancer risk relationship, can promote dietary habits that lower breast cancer risk. The above findings collectively suggest that weight management is the most critical modifiable target in comprehensive lifestyle interventions, and other behavioral changes may exert their protective effects in synergy with weight management.

### 3.8. Chemoprevention

Chemoprevention strategies in this study included tamoxifen and hydroxytyrosol (HT). Pegington et al. [26] studied premenopausal women at moderate‐to‐high breast cancer risk (≥ 17% lifetime risk) not using hormonal contraception, administering tamoxifen (20 mg/day) for 1 year alongside lifestyle recommendations for weight control, alcohol restriction, and increased physical activity. The study concluded that combining these lifestyle measures with medication significantly reduced the incidence of breast cancer and other noncommunicable diseases. But the side effects—including endometrial cancer and thromboembolism—limit its widespread application in women aged ≥ 50 years [34]. Puri et al. [31] evaluated daily oral HT 25 mg for 1 year in premenopausal and postmenopausal women at high risk of breast cancer not receiving standard anti‐estrogen therapy. Although HT did not significantly reduce breast density in the overall population, a significant reduction was observed among women aged ≥ 60 years with high baseline breast density, suggesting that HT may be effective only in specific subgroups. The above evidence indicates that tamoxifen is effective for the overall high‐risk population but has significant side effects, whereas HT has a better safety profile but is effective in a more limited population.

### 3.9. Digital Health Interventions

Digital health interventions leverage digital information technologies to deliver real‐time, individualized (i.e., tailored to individual characteristics such as age, cultural background, health literacy level, and personal preferences) [35], and convenient health behavior promotion to individuals or groups, thereby improving quality of life. Within this category, two subtypes were identified: one is digital behavioral interventions targeting multiple lifestyle behaviors (e.g., diet, physical activity, weight management); the other is digitally delivered health interventions that promote physical activity through digital platforms. Hawkes et al. [32] conducted a qualitative study using semi‐structured interviews with 20 high‐risk women aged 18–35 years with a family history of breast cancer, exploring their experiences with a 2‐month digital behavioral intervention comprising video conferencing, a health education micro‐website, and private social networking groups. Participants generally viewed digital behavioral interventions combining education, tracking, and peer support as a feasible and acceptable approach for managing health behaviors and preventing weight gain in this population. However, they also suggested that digital behavioral interventions need age‐specific individualized tailoring to better meet the needs of women at different life stages [32]. Marinac et al. [21] designed an internet‐based physical activity digital behavioral intervention based on the transtheoretical model (TTM) and SCT. Participants were encouraged to complete 45–60 min of moderate‐to‐vigorous physical activity per week, with activity monitoring, progress charting, peer interaction, and educational content on breast cancer and exercise delivered via an internet platform. Psychologists conducted telephone interviews and sent 2–3 individualized motivational emails per week. Results indicated that women with a family history of breast cancer who received the internet‐based physical activity digital behavioral intervention experienced lifestyle behavior changes facilitated by improved self‐efficacy [21].

The above findings indicate that digital behavioral interventions focus on the synergistic modification of multiple lifestyle behaviors, while digitally delivered physical activity interventions focus on the achievement of a single behavioral target. Both leverage digital platforms to enable real‐time monitoring and individualized feedback for high‐risk women, thereby effectively expanding the reach of preventive interventions.

### 3.10. Health Education and Self‐Management

Several studies suggested that health education and self‐management serve as an important foundational component of breast cancer primary prevention [30]. Zhang [30] developed a primary prevention risk assessment model to enable targeted risk factor modification, incorporating diversified health education, lifestyle guidance, instruction in breast self‐examination, and observation and follow‐up of precancerous lesions. This approach significantly improved women’s self‐care ability and risk awareness, as well as increased voluntary breast cancer screening rates. Deeb et al. [33] further demonstrated that improving women’s knowledge, attitudes, and practices (KAP) regarding breast cancer risk can modify unhealthy dietary and lifestyle habits, cultivate healthy, positive lifestyles and attitudes, and thereby reduce breast cancer risk. The above studies collectively indicate that systematic health knowledge dissemination and skills training can effectively enhance high‐risk women’s awareness of breast cancer risk factors and self‐management capacity, thereby improving unhealthy dietary and lifestyle habits and reducing breast cancer risk.

## 4. Discussion

This scoping review systematically mapped and summarized lower‐cost, nonsurgical primary prevention strategies for women at high risk of breast cancer, incorporating 17 studies across six major categories: nutritional dietary interventions, physical activity interventions, comprehensive lifestyle interventions, chemoprevention, digital health interventions, and health education with self‐management. For the first time, this study provides a reference for breast cancer primary prevention in community and primary healthcare settings from the dual perspectives of “lower‐cost” and “nonsurgical.”

In terms of nutritional dietary intervention, the present evidence reveals two parallel directions: the construction of overall dietary patterns and the supplementation of specific nutrients. Studies have shown that healthy plant‐based diets are associated with reduced breast cancer risk, whereas the effects of specific nutrient supplementation vary across individuals [23]. This finding is consistent with previous research, suggesting that overall dietary patterns may confer more stable protective effects than single‐nutrient supplementation [36]. The individual variability in the effectiveness of nutrient supplementation may be associated with baseline characteristics of study populations (such as genetic susceptibility, alcohol consumption, and menopausal status) [17, 19, 25], suggesting that dietary interventions should consider not only food choices and dietary patterns but also the behavioral and contextual factors that influence how dietary recommendations are adopted and sustained in real‐world settings.

Accordingly, healthcare providers can develop individualized dietary guidance for high‐risk women based on local dietary cultures, age, region, and health literacy levels. For rural or elderly populations, simple dietary mnemonics (e.g., “one jin of vegetables and half a jin of fruit daily, replace white rice with multigrains; consume tofu and soy milk regularly, reduce red and processed meats”) can be used for health education [19, 23, 24, 29]. Local women who have adopted healthy eating habits may be invited to serve as “health ambassadors” for peer education [37]. Additionally, healthcare providers should recognize the impact of alcohol consumption on dietary intervention effects [25], establish health records documenting alcohol intake in high‐risk women, and, for heavy drinkers, provide psychological support or referral to alcohol cessation programs to minimize interference with dietary intervention effects [20, 25], thereby reducing breast cancer risk.

Regarding comprehensive lifestyle intervention, the present study identifies weight management as a central intervention target. Cost‐effectiveness evidence has indicated that lifestyle‐related interventions for breast cancer prevention had a median cost‐effectiveness ratio of $24,973 per quality‐adjusted life year (QALY), with 80% of the interventions falling below the $50,000 willingness‐to‐pay threshold [13]. Therefore, in resource‐limited settings, weight management should be considered as a priority strategy in comprehensive lifestyle interventions. However, the feasibility of weight management interventions in resource‐limited settings may vary depending on local contextual factors, and implementation should be adapted accordingly.

Regarding chemoprevention, tamoxifen was the most commonly used agent in clinical practice in the included studies [34], and its combination with lifestyle interventions significantly reduced the incidence of breast cancer and other noncommunicable diseases in high‐risk women [26]. However, the clinical utility of tamoxifen is tempered by its side effect profile, including increased risks of endometrial cancer and venous thromboembolism in women aged ≥ 50 years, whereas raloxifene offers a more favorable safety profile for postmenopausal women [34]. Aromatase inhibitors demonstrate superior efficacy but are associated with joint pain and bone loss [38].

In terms of cost‐effectiveness, the incremental cost‐effectiveness ratio (ICER) for tamoxifen ranged from $11,530 to $82,504 per QALY gained in women aged < 50 years, those with hysterectomy, and those with high baseline breast cancer risk [34]; the cost‐effectiveness acceptability of aromatase inhibitors was higher over a 12‐year period than over a 5‐year period in the UK and US, but relevant data remain lacking in low‐ and middle‐income countries [39]. Notably, these values are comparable to or higher than the ICERs reported for lifestyle‐based interventions (median $24,973/QALY) [13], suggesting that lower‐cost behavioral strategies may offer favorable value for money from a healthcare system perspective, particularly in resource‐limited settings where pharmaceutical infrastructure is limited.

For patients taking tamoxifen or aromatase inhibitors, healthcare providers should provide thorough premedication education, including information on preventive efficacy, expected treatment duration, common side effects, and management strategies alongside regular follow‐up to monitor adherence and address barriers to continuation [38, 40–42]. Patients with joint stiffness and pain may benefit from warm baths, local heat application, and low‐intensity stretching exercises, which have been shown to alleviate aromatase inhibitor‐associated arthralgia [43–45]. Additionally, attention should be paid to high‐risk women with low health literacy or weak social support, using strategies such as simplified medication regimens, family member involvement in supervision, and community healthcare provider support to enhance long‐term chemoprevention adherence [6].

With respect to digital health interventions and health education with self‐management, this study found that the core advantage of digital approaches lies in transcending temporal and spatial limitations to deliver individualized, lower‐cost, nonsurgical breast cancer prevention strategies. Our findings suggest that digital health interventions are well‐accepted among younger high‐risk women, particularly when incorporating education, tracking, and peer support, but require age‐specific tailoring to maximize engagement [32]. This finding is consistent with previous digital health intervention research, indicating that a one‐size‐fits‐all digital strategy cannot meet the diverse needs of users, and that individualized design is the key to enhancing intervention effectiveness [46]. Healthcare providers and community workers can deliver standardized breast self‐examination training to high‐risk women through home visits, community health fairs, and health education lectures, using illustrated manuals combined with hands‐on demonstrations to explain examination timing, palpation techniques, and precautions, while providing on‐site corrective guidance [47]. Furthermore, internet platforms can be used to regularly disseminate health information and organize online Q&A sessions, forming a continuous care model integrating online and offline approaches and combining digital intervention with humanistic care [48], thereby improving health literacy and reducing breast cancer risk in high‐risk women.

In the domain of health education, available evidence consistently indicates that systematic health knowledge dissemination and skills training can significantly enhance women’s awareness of breast cancer risk factors and self‐management capacity, thereby promoting behavioral and cognitive change and laying a foundation for the implementation of other prevention strategies [30, 33]. The value of health education is particularly evident in low‐ and middle‐income settings, where breast self‐examination represents an early, lower‐cost screening strategy that can be effectively promoted through community‐based education to facilitate early diagnosis [49]. These findings collectively suggest that the cost‐effectiveness of health education is particularly pronounced in populations with low health literacy or in medically underserved areas, and may represent the most scalable foundational prevention strategy in such settings.

### 4.1. Limitations

This study has several limitations. First, considerable heterogeneity in study designs made direct comparisons of prevention strategies difficult. Therefore, future studies may conduct systematic reviews and meta‐analyses to quantitatively synthesize studies of the same design type to enhance comparability of evidence. Second, the majority of included studies were from high‐income countries, with only one from China, necessitating caution in generalizing findings to low‐ and middle‐income countries. Therefore, future research should conduct more empirical studies in low‐ and middle‐income countries to validate the feasibility and acceptability of various prevention strategies across different economic and cultural settings. Third, the search strategy did not include specific terms for particular exercise modalities such as aerobics, yoga, tai‐chi, or pilates, nor for specific dietary patterns such as the Mediterranean diet. Although core keywords such as “exercise,” “physical activity,” and “nutrition” conceptually cover these modalities, studies whose titles or abstracts used only these specific terms may have been missed, and the findings should be interpreted with this limitation in mind. Therefore, future studies may adopt broader search strategies combining specific exercise modalities and dietary pattern keywords with core keywords to capture a more comprehensive body of relevant literature. Fourth, the classification of “lower‐cost” in this study was based primarily on the economic input characteristics of the interventions themselves (e.g., no need for expensive equipment, prolonged hospitalization, or patented drugs), rather than formal economic analyses such as cost‐effectiveness ratios. In addition, this study did not systematically collect detailed cost data for each intervention, nor did it quantify time costs or social costs. Therefore, future research should incorporate health economics evaluation metrics (e.g., ICER, QALY) and conduct quantitative analyses of direct costs, time costs, and social costs to comprehensively assess the true cost‐effectiveness and scalability of each prevention strategy in resource‐limited settings.

### 4.2. Implications for Clinical Practice

The findings provide clear directions for expanding the role of healthcare providers in breast cancer primary prevention. In health education and self‐management, community or breast specialist healthcare providers can deliver breast self‐examination training and individualized health guidance to high‐risk women through home visits, community health fairs, and online communities [50]. Evidence confirms that breast self‐examination is a noninvasive and economically cost‐effective method [51]; compared with clinical breast examination and mammography, it is relatively safe, economically lower‐cost, and independent of healthcare system accessibility [52]. In resource‐limited settings, community health workers delivering health knowledge and skills training can effectively enhance high‐risk women’s awareness of breast cancer risk factors and self‐management capacity [53]. In lifestyle interventions, healthcare providers should provide individualized diet and exercise plans based on age, cultural background, and health literacy levels, such as using digital behavioral interventions for younger women [32] and recommending low‐intensity activities like square dancing and Tai Chi for middle‐aged and elderly women [54]. The World Cancer Research Fund (WCRF) and American Institute for Cancer Research (AICR) cancer prevention recommendations explicitly state that comprehensive lifestyle interventions including maintaining a healthy weight, engaging in regular physical activity (at least 150 min of moderate‐intensity activity per week), consuming whole grains/vegetables/fruits/legumes, and limiting red/processed meats, sugar‐sweetened beverages, and alcohol are core strategies for reducing breast cancer risk [55]. In chemoprevention, healthcare providers should assume core responsibilities for medication adherence management, including premedication education, side effect identification and management, regular follow‐up, and peer support [40, 41]. The U.S. Preventive Services Task Force (USPSTF) recommends that clinicians engage in shared, informed decision‐making with high‐risk women regarding risk‐reducing medications [56]. These clinical practice strategies provide actionable implementation pathways for primary healthcare systems and community care.

### 4.3. Implications for Future Research

Several recommendations for future research emerge from this study. First, standardization of high‐risk population definitions should be promoted, establishing unified risk stratification criteria to facilitate cross‐study integration and comparison. Second, future studies should prioritize the collection and reporting of cost‐effectiveness data. This review found that most original studies did not provide specific cost data for interventions, making judgments of “lower‐cost” based on intervention characteristics rather than precise economic analyses. Subsequent studies are advised to incorporate health economics evaluation metrics (e.g., QALY, ICER) at the design stage to more scientifically assess the scalability of each prevention strategy in resource‐limited settings. Third, more studies are needed in low‐ and middle‐income countries to explore which prevention strategies are most feasible and cost‐effective in resource‐constrained environments. Fourth, long‐term follow‐up studies should be conducted to evaluate the duration of lifestyle intervention effects and dose–response relationships, providing high‐quality evidence for evidence‐based guideline development. Fifth, although digital health interventions show promise, large‐scale RCTs are still needed to validate their effectiveness and explore individualized tailoring to improve adherence.

## 5. Conclusion

This scoping review systematically mapped lower‐cost, nonsurgical primary prevention strategies for women at high risk of breast cancer, identifying six major categories. This study provides reference value for primary healthcare systems, clinical education, and public health policy development. However, due to the heterogeneity of included studies, the concentration of included studies in high‐income countries, and the lack of standardized definitions of high‐risk populations, future research should promote standardization of high‐risk population definitions and further explore these strategies in resource‐limited settings.

## Author Contributions

Menghua Wen: writing–original draft, writing–review and editing, project administration, methodology, investigation, formal analysis, data curation, conceptualization, resources.

Youfu Zhao: methodology, data curation, formal analysis, writing–review.

Chunying He: supervision, funding acquisition, writing–review, project administration.

## Funding

This work was supported by research funds from Research project of Yan’an Bureau of Science and Technology (2023‐SFGG‐055), Yan’an Science and Technology Innovation Team Project (Yan’an Science and Technology Development (2022) No. 41), Science and Technology Development Research Project of Affiliated Hospital of Yan’an University (2022PT‐26) and Natural Science Foundation of Shaanxi Province (2025JC‐YBMS‐835).

## Disclosure

The funder played no role in the study design, data collection and analysis, decision to publish, or manuscript preparation.

## Ethics Statement

Ethical approval was not applicable as no individual patient data or identifiable information was included in this scoping review.

## Consent

Consent was not applicable, as no individual patient data or identifiable information was included in this scoping review.

## Conflicts of Interest

The authors declare no conflicts of interest.

## Supporting Information

Additional supporting information can be found online in the Supporting Information section.

## Supporting information


**Supporting Information 1** The search strategy.

**Supporting Information 2** The PRISMA‐ScR checklist.

**Supporting Information 3** Detailed quality assessment scores of included studies.

## Data Availability

Data are available from the corresponding author upon reasonable request. All data generated or analyzed during this study are included in this published article and its supporting information files.
