# Supplementary material for: Lower‐Cost Nonsurgical Primary Prevention Strategies for Women at High Risk of Breast Cancer: A Scoping Review
Source: Breast J. 2026 Oct 1;2026:1523594. doi: 10.1155/tbj/1523594 (PMC13628707; doi:10.1155/tbj/1523594)
Supplement: Supplementary file 1 [file TBJ-2026-1523594-s001.docx]

**Supplementary Material 1**

**Search Strategy**

**PubMed:**

("Breast Neoplasms"[Mesh] OR "breast cancer*"[tiab] OR "breast carcinoma*"[tiab] OR "mammary cancer*"[tiab] OR "breast tumor*"[tiab] OR "Breast Malignant Neoplasm*") AND (("Risk Factor*"[Mesh] OR "high risk"[tiab] OR "increased risk"[tiab] OR "risk assess*"[tiab] OR "risk management*"[tiab]) AND ("Women"[Mesh] OR "female"[tiab] OR "women"[tiab]))AND ("Primary Prevention"[Mesh] OR "primary prevent*"[tiab] OR "primary prophylax*"[tiab] OR "primary intervent*"[tiab])AND ("life style"[tiab] OR "diet"[tiab] OR "nutrit*"[tiab] OR "exerc*"[tiab] OR "physical activit*"[tiab] OR "health educat*"[tiab] OR "digital intervent*"[tiab] OR "behavioral intervent*"[tiab] OR "chemoprevent*"[tiab] OR "selective estrogen receptor modulator"[tiab] OR "tamoxif*"[tiab] OR "aromatase inhibit*"[tiab] OR "low cost*"[tiab] OR "cost-effect*"[tiab] OR "affordable"[tiab] OR "community-based"[tiab] OR "self-management"[tiab])

**Web of Science:**

TS=("breast cancer*" OR "breast carcinoma*" OR "mammary cancer*" OR "breast tumor*" OR "Breast Malignant Neoplasm*") AND TS=(("risk factor*" OR "high risk" OR "increased risk" OR "risk assess*" OR "risk management*") AND ("female" OR "women")) AND TS=("primary prevent*" OR "primary prophylax*" OR "primary intervent*") AND TS=("life style" OR "diet" OR "nutrit*" OR "exerc*" OR "physical activit*" OR "health educat*" OR "digital intervent*" OR "behavioral intervent*" OR "chemoprevent*" OR "selective estrogen receptor modulator" OR "tamoxif*" OR "aromatase inhibit*" OR "low cost*" OR "cost-effect*" OR "affordable" OR "community-based" OR "self-management")

**EMBASE:**

(‘breast tumor’/exp OR (‘breast cancer*’ OR ‘breast carcinoma*’ OR ‘mammary cancer*’ OR ‘Breast Malignant Neoplasm* OR ‘breast tumo*’ ):ti,ab) AND (‘risk factor’/exp OR (‘risk factor*’ OR ‘high risk’ OR ‘increased risk’ OR ‘risk assess*’ OR ‘risk management*’):ti,ab) AND (‘women’/exp OR (‘female’ OR ‘women’):ti,ab) AND (‘primary prevent’/exp OR (‘primary prevent*’ OR ‘primary prophylax*’ OR ‘primary intervent*’):ti,ab) AND ((‘life style’ OR ‘diet’ OR ‘nutrit*’ OR ‘exerc*’ OR ‘physical activit*’ OR ‘health educat*’ OR ‘digital intervent*’ OR ‘behavioral intervent*’ OR ‘chemoprevent*’ OR ‘selective estrogen receptor modulator’ OR ‘tamoxif*’ OR ‘aromatase inhibit*’ OR ‘low cost*’ OR ‘cost-effect*’ OR ‘affordable’ OR ‘community-based’ OR ‘self-management’):ti,ab)

**Cochrane Library:**

(MeSH descriptor: [Breast Neoplasms] explode all trees OR breast cancer*:ti,ab,kw OR breast carcinoma*:ti,ab,kw OR mammary cancer*:ti,ab,kw OR breast tumor*:ti,ab,kw OR Breast Malignant Neoplasm*:ti,ab,kw) AND (MeSH descriptor: [Risk Factors] explode all trees OR risk factor*:ti,ab,kw OR high risk:ti,ab,kw OR increased risk:ti,ab,kw OR risk assess*:ti,ab,kw OR risk management*:ti,ab,kw) AND (MeSH descriptor: [Women] explode all trees OR female:ti,ab,kw OR women:ti,ab,kw) AND (MeSH descriptor: [Primary Prevention] explode all trees OR primary prevent*:ti,ab,kw OR primary prophylax*:ti,ab,kw OR primary intervent*:ti,ab,kw) AND (life style:ti,ab,kw OR diet:ti,ab,kw OR nutrit*:ti,ab,kw OR exerc*:ti,ab,kw OR physical activit*:ti,ab,kw OR health educat*:ti,ab,kw OR digital intervent*:ti,ab,kw OR behavioral intervent*:ti,ab,kw OR chemoprevent*:ti,ab,kw OR selective estrogen receptor modulator:ti,ab,kw OR tamoxif*:ti,ab,kw OR aromatase inhibit*:ti,ab,kw OR low cost*:ti,ab,kw OR cost-effect*:ti,ab,kw OR affordable:ti,ab,kw OR community-based:ti,ab,kw OR self-management:ti,ab,kw)

**CINAHL(EBSCO):**

(MH "Breast Neoplasms") OR TI (breast cancer* or breast carcinoma* or mammary cancer* or breast tumor* or Breast Malignant Neoplasm*) OR AB (breast cancer* or breast carcinoma* or mammary cancer* or breast tumor* or Breast Malignant Neoplasm*) AND (MH "Risk Factors") OR TI (high risk or increased risk or risk assess* or risk management*) OR AB (high risk or increased risk or risk assess* or risk management*) AND (MH "Women") OR TI (female or women) OR AB (female or women) AND (MH "Primary Prevention") OR TI (primary prevent* or primary prophylax* or primary intervent*) OR AB (primary prevent* or primary prophylax* or primary intervent*) AND TI (life style or diet or nutrit* or exerc* or physical activit* or health educat* or digital intervent* or behavioral intervent* or chemoprevent* or selective estrogen receptor modulator or tamoxif* or aromatase inhibit* or low cost* or cost-effect* or affordable or community-based or self-management) OR AB (life style or diet or nutrit* or exerc* or physical activit* or health educat* or digital intervent* or behavioral intervent* or chemoprevent* or selective estrogen receptor modulator or tamoxif* or aromatase inhibit* or low cost* or cost-effect* or affordable or community-based or self-management)

## **China National Knowledge Infrastructure (CNKI):**

SU=('breast cancer' + 'breast carcinoma' + 'breast tumor' + 'breast neoplasm') AND SU=('risk' + 'harm' + 'hazard' + 'influence' + 'factor') AND SU=('primary' + 'intervention' + 'prevention' + 'chemical' + 'drug' + 'lifestyle' + 'diet' + 'nutrition' + 'exercise' + 'physical activity' + 'health' + 'education' + 'breastfeeding' + 'reproduction' + 'hormone') AND SU=('female' + 'woman' + 'women')

## **Wanfang Database:**

Subject=('breast cancer' OR 'breast carcinoma' OR 'breast tumor' OR 'breast neoplasm') AND Subject=('risk' OR 'harm' OR 'hazard' OR 'influence' OR 'factor') AND Subject=('primary' OR 'intervention' OR 'prevention' OR 'chemical' OR 'drug' OR 'lifestyle' OR 'diet' OR 'nutrition' OR 'exercise' OR 'physical activity' OR 'health' OR 'education' OR 'breastfeeding' OR 'reproduction' OR 'hormone') AND Subject=('female' OR 'woman' OR 'women')

**VIP Database:**

U=('breast cancer' OR 'breast carcinoma' OR 'breast tumor' OR 'breast neoplasm') AND U=('risk' OR 'harm' OR 'hazard' OR 'influence' OR 'factor') AND U=('primary' OR 'intervention' OR 'prevention' OR 'chemical' OR 'drug' OR 'lifestyle' OR 'diet' OR 'nutrition' OR 'exercise' OR 'physical activity' OR 'health' OR 'education' OR 'breastfeeding' OR 'reproduction' OR 'hormone') AND U=('female' OR 'woman' OR 'women')

**China Biomedical Literature Database (CBM):**

Abstract=('breast cancer' OR 'breast carcinoma' OR 'breast tumor' OR 'breast neoplasm') AND Abstract=('risk' OR 'harm' OR 'hazard' OR 'influence' OR 'factor') AND Abstract=('primary' OR 'intervention' OR 'prevention' OR 'chemical' OR 'drug' OR 'lifestyle' OR 'diet' OR 'nutrition' OR 'exercise' OR 'physical activity' OR 'health' OR 'education' OR 'breastfeeding' OR 'reproduction' OR 'hormone') AND Abstract=('female' OR 'woman' OR 'women')

Note: The search terms for the Chinese databases were translated into English for presentation purposes. The actual searches were conducted using the corresponding Chinese terms.
