# Supplementary material for: Lower‐Cost Nonsurgical Primary Prevention Strategies for Women at High Risk of Breast Cancer: A Scoping Review
Source: Breast J. 2026 Oct 1;2026:1523594. doi: 10.1155/tbj/1523594 (PMC13628707; doi:10.1155/tbj/1523594)
Supplement: Supplementary file 3 [file TBJ-2026-1523594-s003.docx]

**Supplementary Materials 3**

# Detailed quality assessment scores of included studie**s**

**Randomized Controlled Trials (RoB 2.0)**

| Study | Randomization Process | Deviations from Intervention | Missing Outcome Data | Outcome Measurement | Overall Bias |
| --- | --- | --- | --- | --- | --- |
| Llanos et al., 2014 | Low | Some concerns | Some concerns | Low | Some concerns |
| Han et al., 2017 | Low | Some concerns | Low | Low | Some concerns |
| Marinc et al.,  2018 | Low | Some concerns | Low | Low | Some concerns |
| Pan et al.,  2019(DM) | Low | Some concerns | Low | Low | Some concerns |
| Marinac  et al.,2018 | Low | Some concerns | Low | Low | Some concerns |

**Cross-Sectional Study (JBI Standard Checklist)**

| Study | Q1 | Q2 | Q3 | Q4 | Q5 | Q6 | Q7 | Q8 | Total |
| --- | --- | --- | --- | --- | --- | --- | --- | --- | --- |
| Deeb et al., 2024 | Yes | Yes | Yes | Yes | Yes | Yes | Yes | Yes | 8 |

**Cohort/Case-Control Study (Newcastle-Ottawa Scale)**

| Study | Selection | Comparability | Outcome | Total |
| --- | --- | --- | --- | --- |
| Peng et al., 2021 | ⭐⭐⭐ | ⭐⭐ | ⭐⭐ | 7 |
| Egnell et al., 2017 | ⭐⭐⭐⭐ | ⭐⭐ | ⭐⭐⭐ | 9 |
| Romanos-Nanclares et al., 2021 | ⭐⭐⭐⭐ | ⭐⭐ | ⭐⭐⭐ | 9 |
| Romanos-Nanclares et al., 2022 | ⭐⭐⭐⭐ | ⭐⭐ | ⭐⭐⭐ | 9 |
| Pegington et al.,  2019 | ⭐⭐⭐⭐ | ⭐ | ⭐⭐⭐ | 8 |
| Vasson et al., 2025 | ⭐⭐⭐⭐ | ⭐⭐ | ⭐⭐ | 8 |
| Shin et al., 2019 | ⭐⭐⭐⭐ | ⭐⭐ | ⭐⭐ | 8 |
| Shin e tal., 2020 | ⭐⭐⭐⭐ | ⭐⭐ | ⭐⭐ | 8 |
| Katsaroli et al., 2024 | ⭐⭐⭐⭐ | ⭐⭐ | ⭐⭐⭐ | 9 |
| Pan et al., 2019(OS) | ⭐⭐⭐⭐ | ⭐⭐ | ⭐⭐⭐ | 9 |

**Single-Arm Studies (MINORS)**

| Study | Q1 | Q2 | Q3 | Q4 | Q5 | Q6 | Q7 | Q8 | Total |
| --- | --- | --- | --- | --- | --- | --- | --- | --- | --- |
| Puri et al., 2025 | 2 | 1 | 2 | 2 | 1 | 2 | 0 | 1 | 11 |

**Quasi-Experimental (MINORS)**

| Study | Q1 | Q2 | Q3 | Q4 | Q5 | Q6 | Q7 | Q8 | Q9 | Q10 | Q11 | Q12 | Total |
| --- | --- | --- | --- | --- | --- | --- | --- | --- | --- | --- | --- | --- | --- |
| Xia et al., 2024 | 2 | 1 | 2 | 2 | 1 | 1 | 2 | 0 | 2 | 2 | 1 | 1 | 17 |

**Qualitative Study (JBI-QARI)**

| Study | Q1 | Q2 | Q3 | Q4 | Q5 | Q6 | Q6 | Q7 | Q8 | Q9 | Q10 | Total |
| --- | --- | --- | --- | --- | --- | --- | --- | --- | --- | --- | --- | --- |
| Hawkes  et al., 2024 | Yes | Yes | Yes | Yes | Yes | Yes | Yes | Yes | Yes | Yes | Yes | A |
